# MeHg-induced autophagy via JNK/Vps34 complex pathway promotes autophagosome accumulation and neuronal cell death

**DOI:** 10.1038/s41419-019-1632-z

**Published:** 2019-05-21

**Authors:** Tianji Lin, Shijuan Ruan, Dingbang Huang, Xiaojing Meng, Wenjun Li, Bin Wang, Fei Zou

**Affiliations:** Department of Occupational Health and Occupational Medicine, School of Public Health, Southern Medical University, 510515 Guangzhou, Guangdong China

**Keywords:** Macroautophagy, Stress signalling, Cell death in the nervous system

## Abstract

Methylmercury (MeHg), an environmental toxin, may specifically cause neurological disorders. Recent studies have reported that autophagy can be induced by metals and be involved in metal cytotoxicity. However, the role of autophagy in MeHg-induced neurotoxicity remains unknown. Here, we demonstrate that MeHg induces mTOR-independent autophagy through JNK/Vps34 complex pathway, which further promotes autophagosome accumulation and neuronal cell death. In addition to cell death, MeHg increased LC3-II expression in a concentration- and time-dependent manner in neuronal cells; furthermore, western blot analysis of LC3-II expression under baf A1-treated condition indicates that MeHg activates autophagy induction. However, we found lysosomal degradative function was impaired by MeHg. Under this condition, MeHg-activated autophagy induction would elicit autophagosome accumulation and cell death. Consistent with this inference, the autophagy inhibitor decreased the MeHg-induced autophagosome accumulation and neuronal cells death, whereas the autophagy inducers further augmented MeHg cytotoxicity. Then, the mechanism of autophagy induction is investigated. We show that MeHg-induced autophagy is mTOR-independent. Vacuolar protein sorting 34 (Vps34) complex is critical for mTOR-independent autophagy. MeHg induced the interaction between Beclin1 and Vps34 to form Vps34 complex. Importantly, knockdown of Vps34 inhibited autophagy induction by MeHg. Furthermore, we found that JNK, but not p38 or ERK, promoted the formation of Vps34 complex and autophagy induction. Finally, inhibition of JNK or downregulation of Vps34 decreased autophagosome accumulation and alleviated MeHg-induced neuronal cell death. The present study implies that inhibiting JNK/Vps34 complex autophagy induction pathway may be a novel therapeutic approach for the treatment of MeHg-induced neurotoxicity.

## Introduction

Mercury has been continually discharged from natural sources as well as industrial activities in recent years^1^. Microorganisms are able to transform inorganic mercury to MeHg, then MeHg accumulates in plants and animals. In this way, MeHg reaches the food chain, which is dangerous for humans^2^. MeHg can cross the blood–brain barrier to accumulate in the central nervous system (CNS)^3^. MeHg induces neurotoxic effects that lead to developmental deficits such as loss of intelligence quotient (IQ) points, reduction in language skills, impaired memory function, and attention deficits in children exposed in-utero^4^.

Several studies have reported that autophagy can be induced by starvation, nutrient depletion, and environmental stress^5,6^. Under physiological condition, autophagy protects cell from death through sequestering damaged protein and organelles into autophagosomes formed by autophagy induction and delivering autophagosomes to lysosomes for degradation. In the studies on the neurotoxicity of certain metal (such as cadmium and manganese), autophagy can protect neuronal cell from death^7,8^. However, autophagy induction can also serve a pro-death role in pathological conditions. In vivo and in vitro, studies have reported that autophagy induction promotes cytotoxicity through excessive elimination of intracellular organelles and cytosol^9,10^ or autophagosome accumulation^11–14^. What role autophagy plays in MeHg-induced neurotoxicity remains unclear.

Autophagy is induced by mTOR-dependent or mTOR-independent pathway^15^. The mTOR-dependent autophagy is negatively regulated by mTOR^16^. In addition to nutrient starvation, the neurotoxin has been found to induce autophagy via mTOR-dependent pathway^17^. In mTOR-independent autophagy, Vps34 complex is a critical mediator for autophagy induction^18^. Beclin1 binds to Vps34 to form the core Vps34 complex to activate autophagy induction. It is not known whether the MeHg-induced autophagy is mTOR-dependent or mTOR-independent.

MAPKs (mitogen-activated protein kinases) have been reported to be related to autophagy induction and play an important role in neurotoxicity^19–21^. The mammalian MAPK family consists of ERK (extracellular signal-regulated kinase), p38, and JNK (c-Jun NH2-terminal kinase). Various observations suggest that MAPKs contribute to metals-induced neurotoxicity^22,23^. However, how MAPKs regulate autophagy induction and cell death in MeHg-induced neurotoxicity are not understood.

In this study, we demonstrate that MeHg induces mTOR-independent autophagy through JNK/Vps34 complex pathway, which further promotes autophagosome accumulation and neuronal cell death.

## Results

### MeHg induces neuronal cell death

**Fig. 1:**
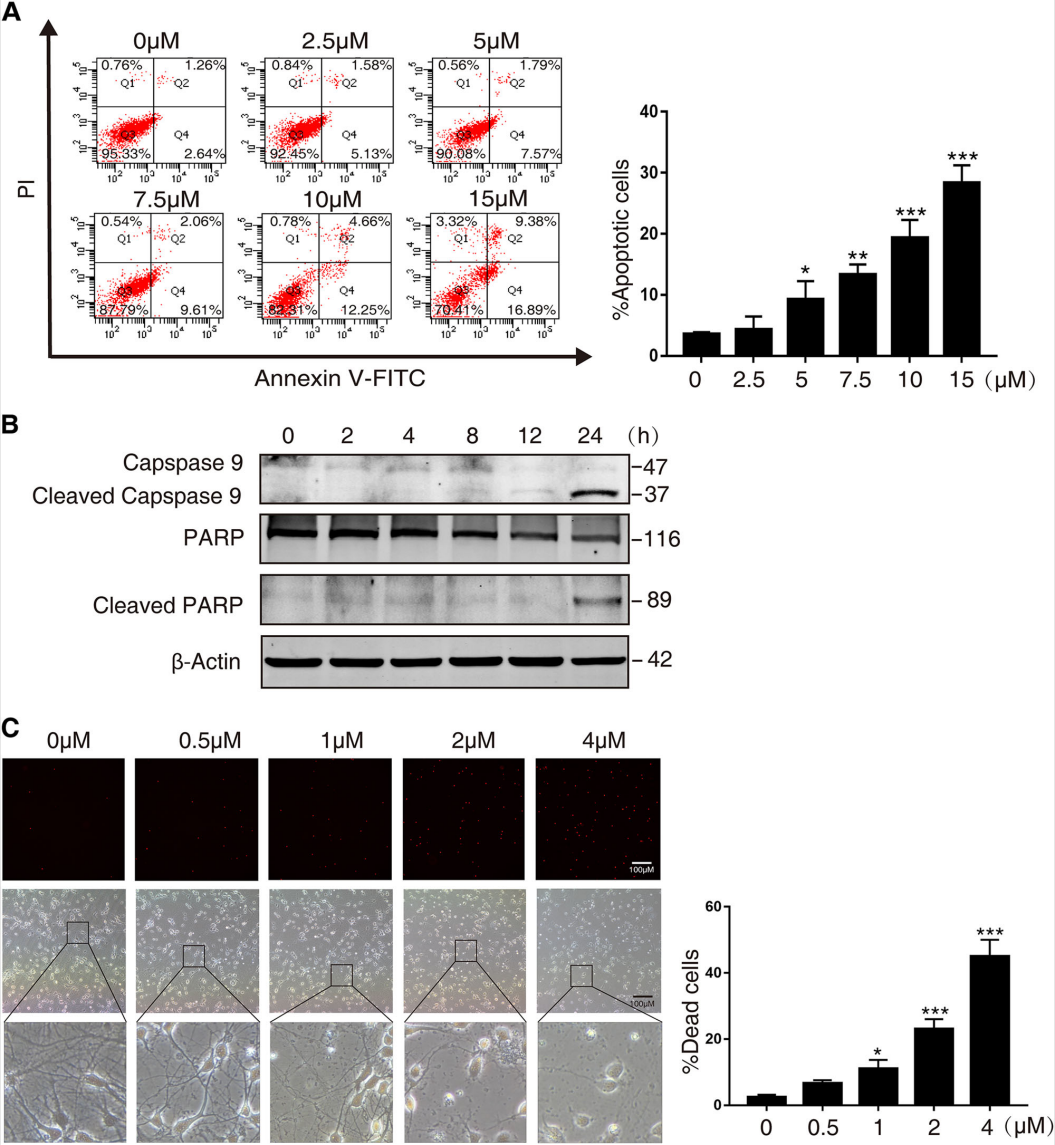
MeHg induces neuronal cell death. **a** SH-SY5Y cells were treated with indicated concentrations of MeHg for 24 h. Cell apoptosis was detected using an annexin V FITC/PI apoptosis detection kit and flow cytometry analysis. *P < 0.05 versus 0 µM MeHg group, **P < 0.01 versus 0 µM MeHg group, ***P < 0.001 versus 0 µM MeHg group. **b** SH-SY5Y cells were exposed to 10 µM MeHg at indicated times and then harvested. The cell lysates were subjected to western blot analysis to measure caspase-9 and PARP and their cleaved fragments expression. β-actin was used as an internal control. **c** Rat cerebral cortical neurons were treated with indicated concentrations of MeHg for 12 h and stained with propidium iodide (PI) to detect cell death via fluorescent microscopy. *P < 0.05 versus 0 µM MeHg group, ***P < 0.001 versus 0 µM MeHg group. All results are representative of three independent experiments

We used SH-SY5Y cells and rat cerebral cortical neurons to study MeHg-induced neuronal cell death. As shown in Fig. 1a, treatment with MeHg for 24 h caused a concentration-dependent increase of apoptosis in SH-SY5Y cells. Furthermore, MeHg increased cleaved fragments of caspase-9 and PARP (apoptosis markers) at 24 h (Fig. 1b). Moreover, in rat cerebral cortical neurons, MeHg induced cell death and impaired synapse in a dose-dependent manner at 12 h (Fig. 1c). These results indicate that MeHg induces neuronal cell death.

### MeHg increases autophagy induction in neuronal cells

**Fig. 2:**
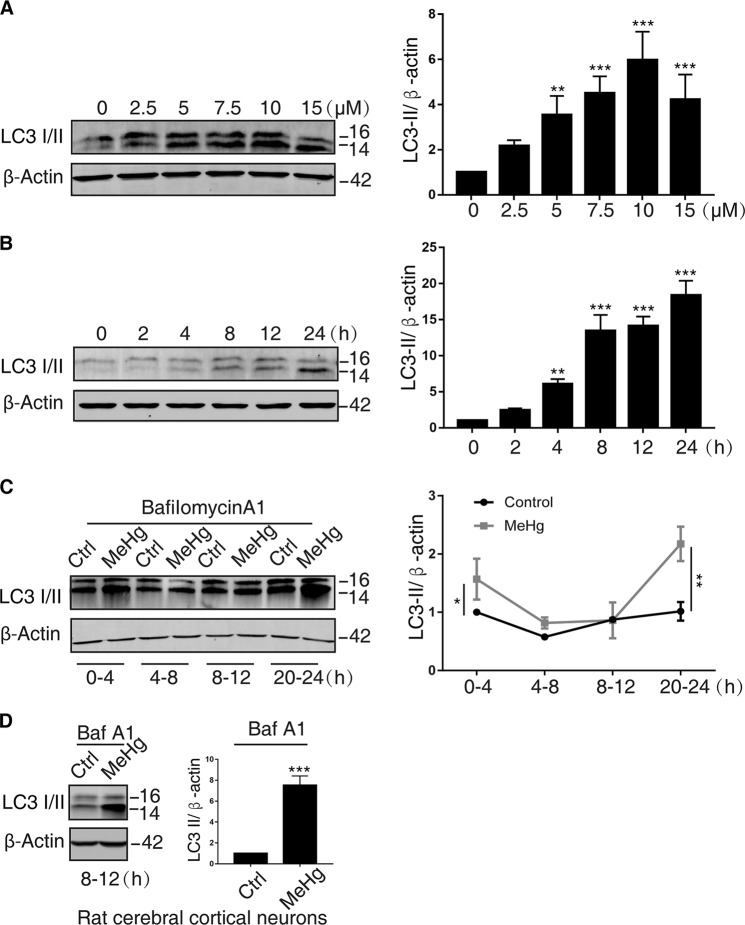
MeHg induces LC3 protein expression in both SH-SY5Y cells and rat cerebral cortical neurons. **a** SH-SY5Y cells were treated with indicated concentrations of MeHg for 24 h and then harvested. The cell lysates were subjected to western blot analysis to measure LC3-II expression. **P < 0.01 versus 0 µM MeHg group, ***P < 0.001 versus 0 µM MeHg group. **b** SH-SY5Y cells were exposed to 10 µM MeHg at indicated times and then harvested. The cell lysates were subjected to western blot analysis to measure LC3-II expression. **P < 0.01 versus 0 µM MeHg group, ***P < 0.001 versus 0 µM MeHg group. **c** MeHg-exposed (10 µM) SH-SY5Y cells were treated with bafilomycin A1 (baf A1; 400 nM) for 4 h prior to sample processing at different time point. The cell lysates were subjected to western blot analysis to measure LC3-II expression. *P < 0.05 versus 0–4 h control group, **P < 0.01 versus 20–24 h control group. **d** MeHg-exposed (2 µM) rat cerebral cortical neurons were treated with bafilomycin A1 (baf A1; 400 nM) for 4 h prior to sample processing at 12 h. The cell lysates were subjected to western blot analysis to measure LC3-II expression. ***P < 0.001 versus 8–12 h control group. All results are representative of three independent experiments

Autophagy is known as one of the critical cellular homeostatic mechanisms and closely related to cell death. LC3-II is the most commonly used marker of autophagy induction^24^. As shown in Fig. 2a, b, MeHg induced LC3-II protein expression in the concentration- and time-dependent manner in SH-SY5Y cells. To further determine whether MeHg activated autophagy induction, bafilomycin A1 (baf A1) was used to inhibit autophagic degradation for 4 h before sample collection. We found that MeHg induced LC3-II expression more significantly compared with control group during 0–4 h and 20–24 h under baf A1-treated condition (Fig. 2c). Similar results were observed in baf A1-treated rat cerebral cortical neurons during 8–12 h after MeHg exposure (Fig. 2d). These findings suggest that MeHg increases autophagy induction in neuronal cells.

### Autophagy induction promotes autophagosome accumulation and cell death in MeHg-exposed neuronal cells

**Fig. 3:**
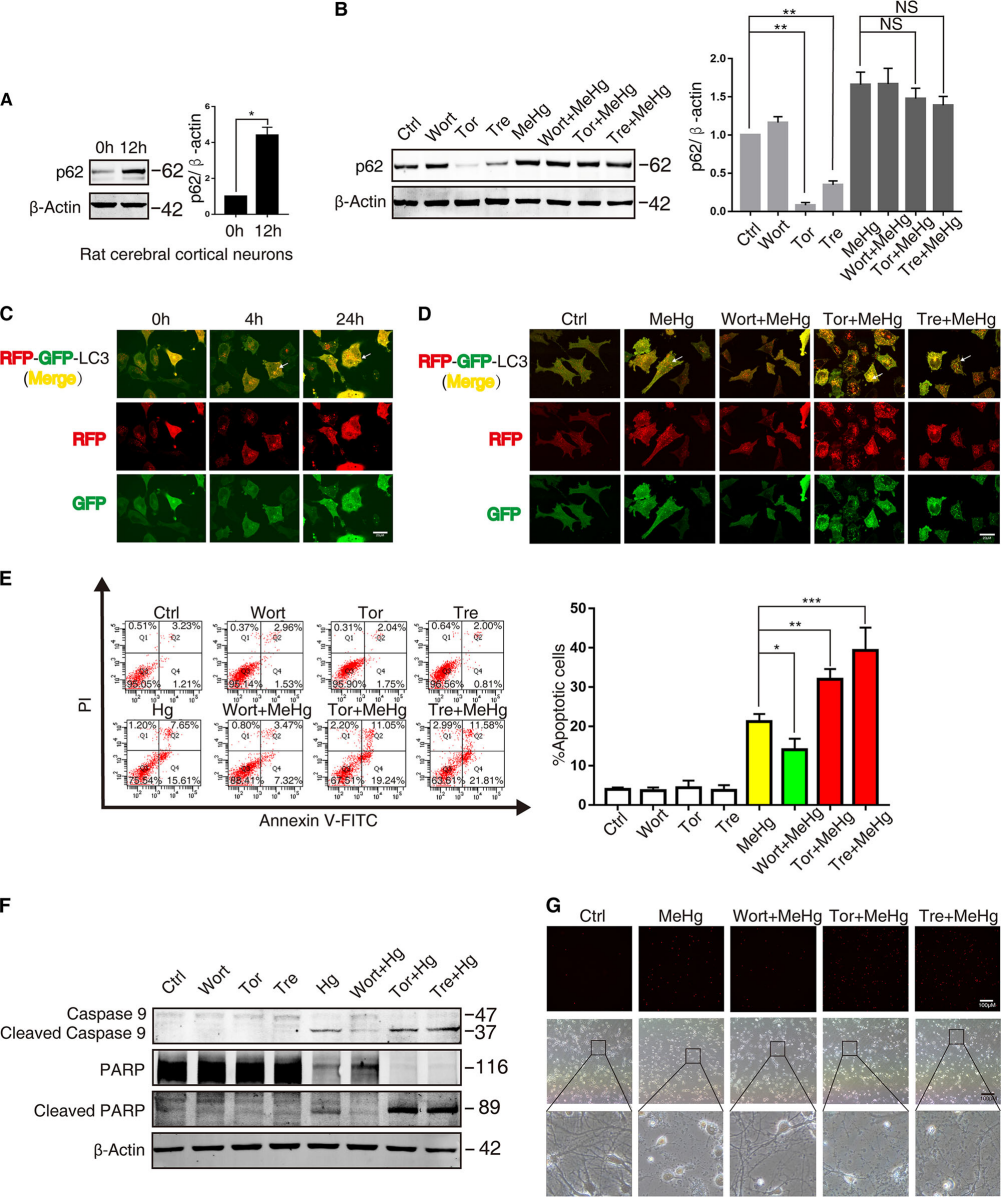
MeHg leads to autophagosome accumulation and neuronal cell death. **a** Rat cortical neurons were treated with 2 µM MeHg for 12 h. The cell lysates were subjected to western blot analysis to measure p62 expression. *P < 0.05 versus 0 h group. **b** SH-SY5Y cells were pretreated with wortmannin (wort; 1 µM 1 h), Torin1 (Tor; 1 µM 1 h), or trehalose (tre; 50 mM 1 h), then the cells were exposed to 10 µM MeHg for 24 h. The cell lysates were subjected to western blot analysis to measure p62 expression. **P *<* 0.01 versus control group, NSå 0.05 versus MeHg group. **c** SH-SY5Y cells were infected with adeno-associated viral vector (AAV-mRFP-GFP-LC3) at final MOI 100 for 24 h. Then cells were exposed to 10 µM MeHg at indicated times. The cells were examined by confocal microscopy (×100). **d** After AAV-mRFP-GFP-LC3 infection, the protocol was followed as described in **b**. Then the SH-SY5Y cells were examined by confocal microscopy (×100). **e** The protocol was followed as described in **b**. Cell apoptosis was detected using an annexin V FITC/PI apoptosis detection kit and flow cytometry analysis. *P < 0.05 versus MeHg group, **P < 0.01 versus MeHg group, ***P < 0.001 versus MeHg group. **f** The protocol was followed as described in **b**. The cell lysates were subjected to western blot analysis to measure caspase-9 and PARP and their cleaved fragments expression. **g** Rat cerebral cortical neurons were pretreated with wortmannin (wort; 100 nM 1 h), Torin1 (Tor; 250 nM 1 h), or trehalose (tre; 50 mM 1 h), then the cells were exposed to 2 µM MeHg for 12 h and stained with propidium iodide (PI) to detect cell death via fluorescent microscopy. All results are representative of three independent experiments

Once stress activates autophagy induction to form autophagosome, the following efficient degradation of autophagy substrates (protein aggregates and damaged organelles) is critical for maintaining cellular homeostasis^25^. To ascertain the role of autophagy induction in MeHg-induced cell death, lysosomal degradative function was therefore determined through western blot analysis of p62 protein expression. An increase in p62 was observed in cerebral cortical neurons treated with 2 µM MeHg for 12 h (Fig. 3a). Moreover, the autophagic inducers Torin1 (1 µM) and trehalose (5 mM) decreased p62 protein expression more markedly in control group compared with that in MeHg-treated SH-SY5Y cells (Fig. 3b). These results indicate that MeHg impairs the autophagic degradation, and under this condition, increased autophagy induction would elicit autophagosome accumulation.

To further investigate the effect of autophagy induction on autophagosome accumulation, SH-SY5Y cells were pretreated with autophagy inhibitor or inducer, respectively. The AAV-mRFP-GFP-LC3 introduced into neuronal cells was used to detect autophagosome accumulation^26,27^. MeHg increased the number and the size of the yellow puncta (autophagosomes) in SH-SY5Y cells at 4 h and 24 h, which indicates that MeHg induces autophagosome accumulation (Fig. 3c). Importantly, wortmannin (autophagy inhibitor) decreased the number of autophagosomes in MeHg-treated SH-SY5Y cells, whereas Torin1 or trehalose (autophagy inducer) further increased the number and size of the autophagosomes (Fig. 3d). These results suggest that MeHg-induced autophagy promotes autophagosome accumulation.

Finally, neuronal cells were pretreated with autophagic inhibitor or inducer to further ascertain the role of MeHg-induced autophagy in neuronal cell death. Wortmannin decreased the apoptosis in MeHg-treated SH-SY5Y cells, whereas Torin1 and trehalose augmented MeHg-induced apoptosis (Fig. 3e). Furthermore, the cleaved fragments of caspase-9 and PARP were decreased by wortmannin and increased by Torin1 or trehalose (Fig. 3f). In rat cerebral cortical neurons, the effects of autophagic inhibitor or inducer on MeHg-induced cytotoxicity were similar to those observed in the SH-SY5Y cells (Fig. 3g). These findings indicate that MeHg-activated autophagy induction promotes autophagosome accumulation and neuronal cell death.

### MeHg induces mTOR-independent autophagy through JNK/Vps34 complex signaling pathway

**Fig. 4:**
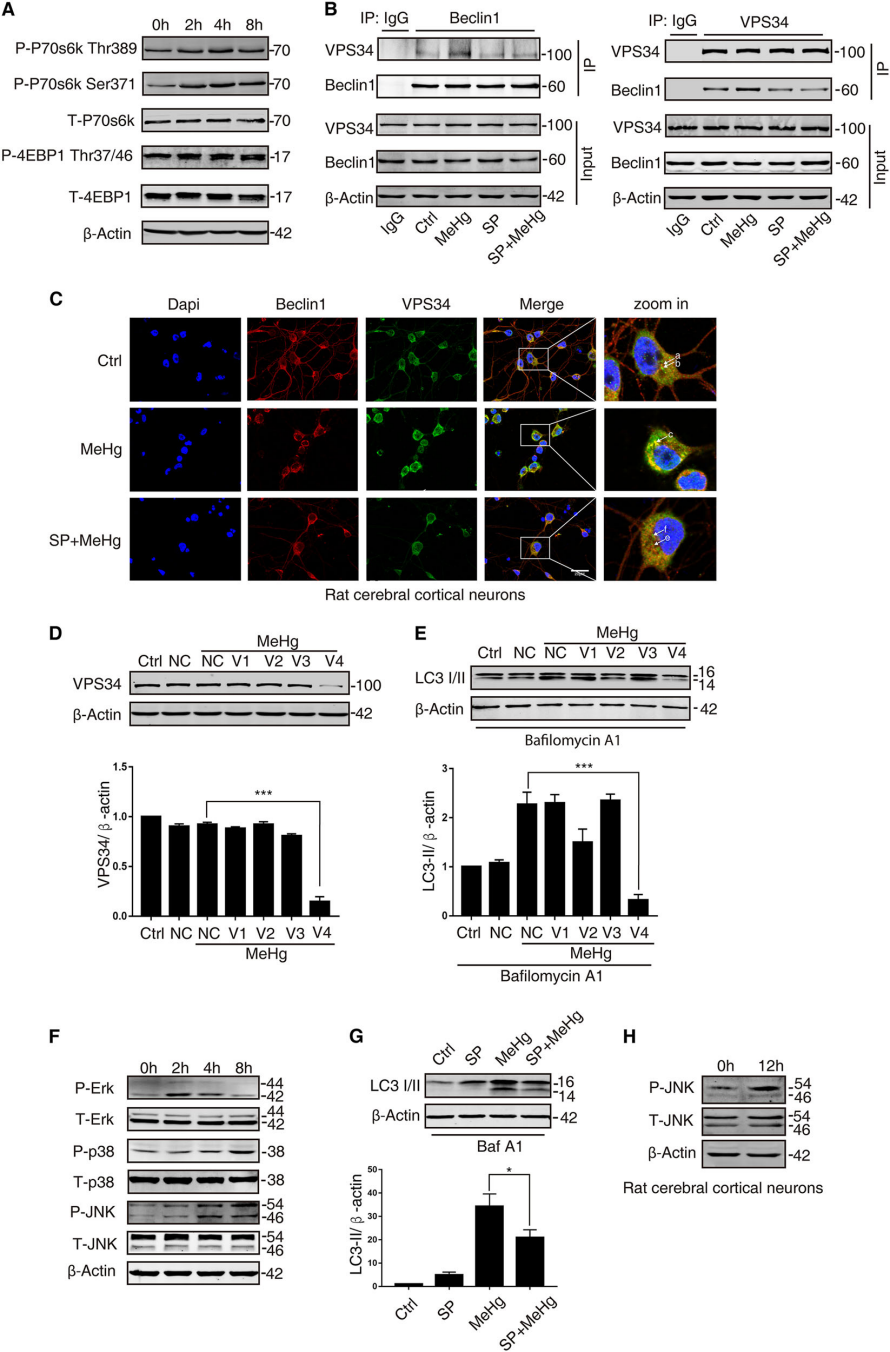
MeHg induces mTOR-independent autophagy through JNK/Vps34 complex signaling pathway. **a** SH-SY5Y cells were exposed to 10 µM MeHg at indicated times. Phosphorylated and total mTOR substrate were detected by western blot. **b** SH-SY5Y cells were exposed to 10 µM MeHg individually for 4 h or in combination with pretreatment of 30 µM SP600125 for 1 h. The lysates were co-immunoprecipitated using a Beclin1 (Vps34)-specific antibody, and the immunoprecipitants (pull down) were analysed by western blot for the presence of Vps34(Beclin1). The whole cell lysates (input) were analysed by western blot for Vps34 and Beclin1. **c** Rat cerebral cortical neurons were exposed to 2 µM MeHg individually for 12 h or in combination with pretreatment 2 µM SP600125 for 1 h. The cells were stained to determine the colocalization of Beclin1 (red) and Vps34 (green). The cell nucleus was stained with DAPI (blue). **d** Negative control (NC) siRNA and Vps34 siRNAs (V1–V4) were transiently transfected into SH-SY5Y cells for 24 h. Then, the cells were exposed to 10 µM MeHg for 4 h. Western blot analysis was performed to determine the Vps34 protein level. ***P < 0.001 versus MeHg-exposed negative control group. **e** The transfected cells exposed to MeHg were treated with bafilomycin A1 (baf A1, 400 nM) for 4 h before sample processing. LC3-II was detected by western blot. ***P < 0.001 versus MeHg-exposed negative control group. **f** SH-SY5Y cells were exposed to 10 µM MeHg at indicated times and then harvested. Phosphorylated and total MAPK were detected by western blot. **g** SH-SY5Y cells were exposed to 10 µM MeHg for 24 h after preincubation with JNK inhibitor 30 µM SP600125 for 1 h. Then, the cells were treated with bafilomycin A1 (baf A1, 400 nM) for 4 h before sample processing. LC3-II was detected by western blot. *P < 0.05 versus MeHg group. **h** Rat cerebral cortical neurons were exposed to 10 µM MeHg for 12 h. Phosphorylated and total JNK were detected by western blot. All results are representative of three independent experiments

Autophagy induction signaling involves mTOR-dependent and mTOR-independent pathways^15^. The mTOR-dependent autophagy is negatively regulated by mTOR. mTOR activity can be inferred by the levels of phosphorylation of its substrates (p70S6K and 4E-BP1)^28^. As shown in Fig. 4a, MeHg increased phosphorylation of p70S6K (Thr389, Ser371) and 4E-BP1 (Thr 37/46). Thus, MeHg induces mTOR-independent autophagy.

Vps34 has been reported to activate mTOR-independent autophagy induction through binding Beclin1 to form the Vps34 core complex^18^. To determine whether MeHg induced interaction between Beclin1 and Vps34, Co-immunoprecipitation and immunofluorescence experiment were performed. As shown in Fig. 4b, MeHg enhanced the formation of Beclin1-Vps34 complex in SH-SY5Y cells. Consistently, MeHg increased Beclin1 (Fig. 4c, arrow a) and Vps34 (Fig. 4c, arrow b) colocalization (Fig. 4c, arrow c) detected by immunofluorescence in rat cerebral cortical neurons. To further address the role of Vps34 core complex in autophagy induction, chemically synthesized siRNAs against Vps34 were introduced into SH-SY5Y cells. Four siRNAs were screened and western blot analysis showed siRNA-1, siRNA-2, or siRNA-3 had little effect on downregulating Vps34 protein expression. However, Vps34 protein levels in SH-SY5Y cells transfected with siRNA-4 were reduced to 10% of that found in cultures transfected with the NC siRNA (Fig. 4d). Importantly, transfection with siRNA-4 reduced LC3-II induction in MeHg-exposed SH-SY5Y cells (Fig. 4e). These findings suggest that MeHg increases mTOR-independent autophagy induction through enhancing forming the Beclin1-Vps34 complex in neuronal cells.

MAPKs have been reported to be involved in mTOR-independent autophagy^29–31^. Therefore, MAPKs activation was investigated following MeHg treatment. We found that MAPKs (ERK, p38, and JNK) were activated at different time by MeHg in SH-SY5Y cells (Fig. 4f). To further assess the effects of MAPKs on the VPS34 complex-dependent autophagy induction, SH-SY5Y cells were exposed to MeHg after pretreatment with MEK (upstream kinase of ERK) inhibitor U0126, p38 inhibitor SB203580, or JNK inhibitor SP600125 for 1 h. Notably, pretreatment with SP600125 caused a dissociation of the Beclin1 and Vps34 complex (Fig. 4c, arrow e and f) and reduced LC3-II induction in MeHg-exposed SH-SY5Y cells (Fig. 4g). However, pretreatment with U0126 and SB203580 had little effect on VPS34 complex formation and LC3-II induction (Fig S1 a, b). Moreover, in rat cerebral cortical neurons, JNK was also activated with 2 µM MeHg for 12 h (Fig. 4h). Immunofluorescence results showed that pretreatment with SP600125 disrupted the MeHg-induced colocalization between Beclin1 and Vps34. Thus, MeHg increases mTOR-independent autophagy induction through activating the JNK/Vps34 complex signaling pathway in neuronal cells.

### Downregulating JNK/Vps34 complex autophagy induction pathway can reduce the autophagosome accumulation and alleviates the neuronal cell death induced by MeHg

**Fig. 5:**
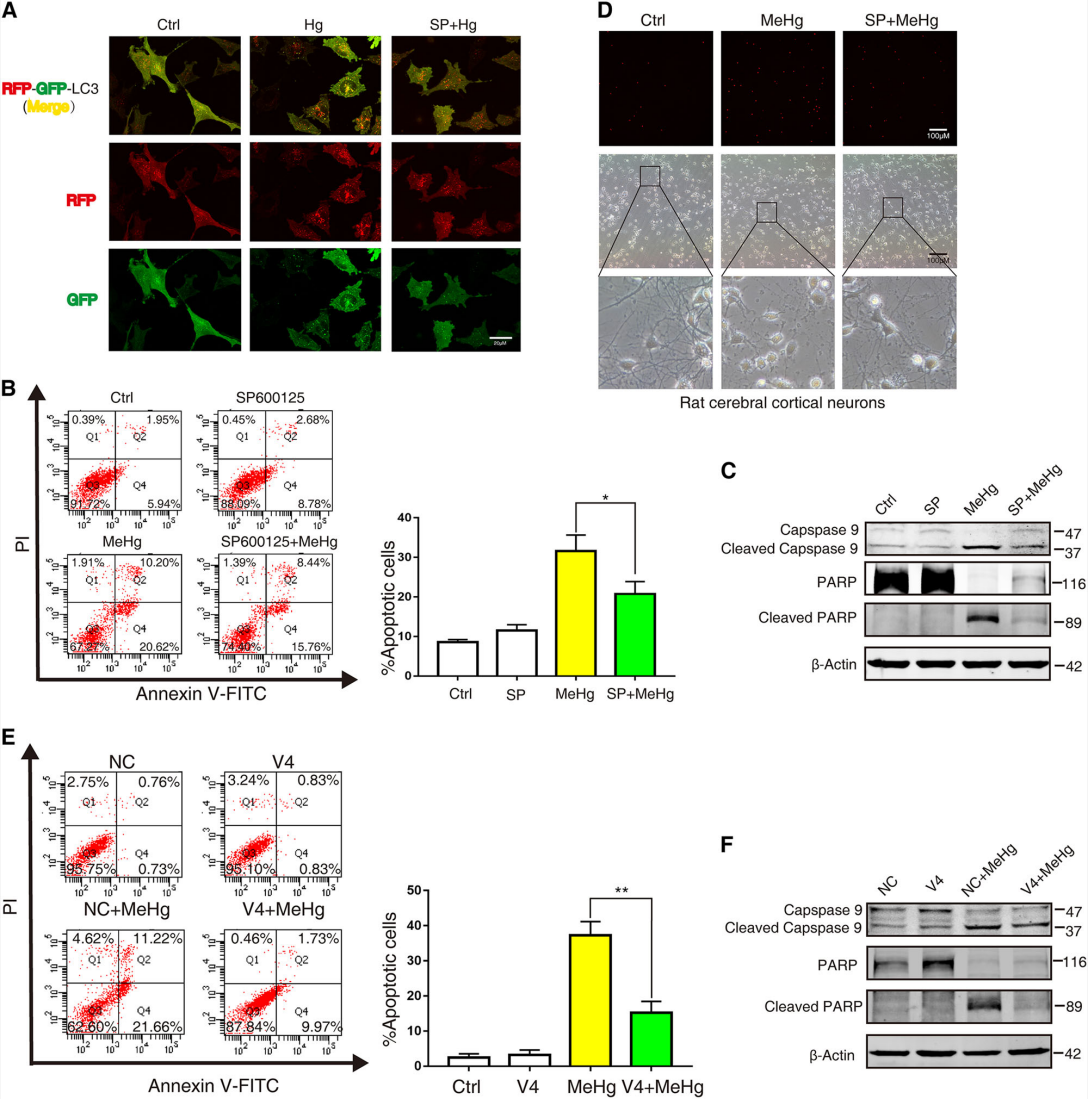
Downregulating JNK/Vps34 complex autophagy induction pathway alleviated MeHg-induced neuronal cell death. **a** SH-SY5Y cells were infected with adeno-associated viral vector (AAV-mRFP-GFP-LC3) at final MOI 100 for 24 h. Then cells were exposed to 10 µM MeHg for 4 h after preincubation with JNK inhibitor 30 µM SP600125 for 1 h. Finally, the cells were examined by confocal microscopy (×100). **b** SH-SY5Y cells were exposed to 10 µM MeHg for 24 h after preincubation with JNK inhibitor 30 µM SP600125 for 1 h. Cell apoptosis was detected with an annexin V FITC/PI apoptosis detection kit using flow cytometry analysis. *P < 0.05 versus MeHg group. **c** The protocol was followed as described in **b**, Caspase-9 and PARP and their cleaved fragments expression were detected by western blot. **d** Rat cerebral cortical neurons were exposed to 2 µM MeHg for 12 h after preincubation with JNK inhibitor 2 µM SP600125 for 1 h. Cells stained with propidium iodide (PI) to detect cell death via fluorescent microscopy. **e** After transfection with NC siRNA and Vps34 siRNA-4 for 24 h, the SH-SY5Y cells were exposed to 10 µM MeHg for 24 h. Cell apoptosis was detected using an annexin V FITC/PI apoptosis detection kit and flow cytometry analysis. **P < 0.01 versus 0 µM MeHg group. **f** The protocol was followed as described in **e**, Caspase-9 and PARP and their cleaved fragments expression were measured by western blot. All results are representative of three independent experiments

To further determine the effects of JNK/Vps34 complex pathway on autophagosome accumulation and neuronal cell death, neuronal cells were pretreated with SP600125 or transfected with siRNA. As shown in Fig. 5a, SP600125 decreased autophagosome accumulation (yellow puncta) in MeHg-exposed SH-SY5Y cells. Correspondingly, pretreatment with SP600125 suppressed the apoptosis and decreased the cleaved fragments of caspase 9 and PARP induced by MeHg in SH-SY5Y cells (Fig. 5b, c). However, pretreatment with U0126 and SB203580 had little effect on suppressing the MeHg-induced apoptosis of SH-SY5Y cells (Fig S2). Similar to results in SH-SY5Y cells, pretreatment with SP600125 suppressed cell death and synapse breakage induced by MeHg in rat cerebral cortical neurons (Fig. 5d). Moreover, transfection with siRNA against Vps34 also reduced MeHg-induced apoptosis and decreased the cleaved fragments of caspase 9 and PARP in MeHg-exposed SH-SY5Y cells. (Fig. 5e, f). Taken together, these findings suggest that downregulating JNK/Vps34 complex autophagy induction pathway can reduce the autophagosome accumulation and alleviate the neuronal cell death induced by MeHg.

## Discussion

MeHg, a toxic heavy metal, may cause neurological disorders. In the current study, MeHg induced a concentration-dependent increase of cell death in SH-SY5Y cells and rat cerebral cortical neurons. In addition to cell death, certain metals, such as cadmium and manganese can induce autophagy in neuronal cells^32–34^. Autophagy induction had been found to be activated in MeHg-treated human neural stem cells^35^. In the current study, MeHg increased LC3-II protein expression in a concentration- and time-dependent manner in neuronal cells; furthermore, western blot analysis of LC3-II protein expression under baf A1-treated condition showed that autophagy induction increased at 4 h and 24 h after MeHg exposure. These findings suggest that MeHg induces neuronal cell death and activates autophagy induction.

There is a close relationship between autophagy and cell death^36^. Through autophagy induction and autophagic degradation of damaged protein and organelle, autophagy can alleviate cell injury. It has been reported that autophagy plays a protective role in metals (such as cadmium and manganese)-induced neurotoxicity^7,8^. In the current cell model, after MeHg activates autophagy induction to form autophagosome, efficient degradation of autophagy substrates is essential for alleviation of MeHg-induced neurotoxicity. However, we found that MeHg increased p62 expression that is the index used for detection of lysosomal degradative function^37^, and the autophagic inducers could decrease p62 expression in normal control cells, but this effect was abated in MeHg-treated cells; moreover, MeHg induced lysosomal membrane permeabilization (LMP) (data not shown). These results indicate that lysosomal degradative function is impaired by MeHg. Under this condition, increased autophagy induction would elicit excessive autophagosome accumulation^11–14^. Consistent with this inference, in the current study, we found that activating or inhibiting the autophagy induction by pharmacological methods increased or decreased autophagosome accumulation in MeHg-exposed neuronal cells. More importantly, activating or inhibiting the autophagy induction had the corresponding effect on cell death. Thus, increased autophagy induction by MeHg promotes autophagosome accumulation and the neuronal cell death. Several studies have reported that autophagosome accumulation is detrimental to cells under stress and pathological situations^38,39^. Accumulation of autophagosomes could result in overproduction of ROS. Moreover, the energy deficit resulting from impaired autophagic degradation also contributes to autophagosome accumulation-induced cytotoxicity^40^. It seems that autophagosome accumulation elicits cell death via different mechanisms, which needs further study.

Autophagy induction is regulated by mTOR-dependent pathway or mTOR-independent pathway^15^. However, the pathway which regulates the MeHg-induced autophagy is still unknown. The mTOR- dependent autophagy is negatively regulated by mTOR^16^. Our results showing that mTOR substrates were activated in MeHg-treated neuronal cells suggest that the MeHg-induced autophagy is mTOR-independent.

It has been reported that the Vps34 complex is critical for autophagy induction in mTOR-independent autophagy. Beclin1 binds to Vps34 to form Vps34 complex and start autophagy^18^. By combined use of co-immunoprecipitation and immunofluorescence technology, we show for the first time that MeHg enhances the Beclin1-Vps34 complex formation. Furthermore, chemically synthesized siRNAs against Vps34 inhibited autophagy induction. These results indicate that MeHg increases mTOR-independent autophagy induction through enhancing forming the Vps34 complex in neuronal cells.

MAPKs can regulate mTOR-independent autophagy^15,30,41^ and are reported to play an important role in MeHg-induced neurotoxicity^42,43^. We found that MAPKs (JNK, p38, and ERK) were activated by MeHg in neuronal cells. JNK, but not p38 or ERK, promotes the interaction between Beclin1 and Vps34 and autophagy induction in MeHg-treated neuronal cells. It is unclear how JNK regulates the formation of Vps34 complex. Further studies are needed to address this issue. Moreover, Inhibition of JNK or downregulation of Vps34 can decrease autophagosome accumulation and alleviate MeHg-induced neuronal cell death.

Several reports have suggested that calcium dyshomeostasis^44,45^, oxidative stress^46,47^, and disruption of the cytoskeleton^48^ are important mechanisms accounting for MeHg-induced neurotoxicity. How do these mechanistic signals regulate JNK-activated autophagy induction? It has been reported that Ca^2+^ signal and reactive oxygen species (ROS) may mediate sustained JNK activation through CaMKII^49–51^ and ASK1^52,53^, respectively, and Ca^2+^ signal, ROS, and phagophore membranes origination can be found at endoplasmic reticulum (ER)-mitochondria contact sites^51,54,55^. These reports raise the possibility that local Ca^2+^ signal and ROS might regulate JNK-activated autophagy induction and be involved in MeHg-induced neurotoxicity. Moreover, calcium dyshomeostasis and oxidative stress may contribute to impaired autophagic degradation. High Ca^2+^ signaling inhibits the autophagosome-lysosome fusion^56,57^ and oxidative stress leads to lysosomal dysfunction^58,59^. These potential mechanisms need further extensive study.

**Fig. 6:**
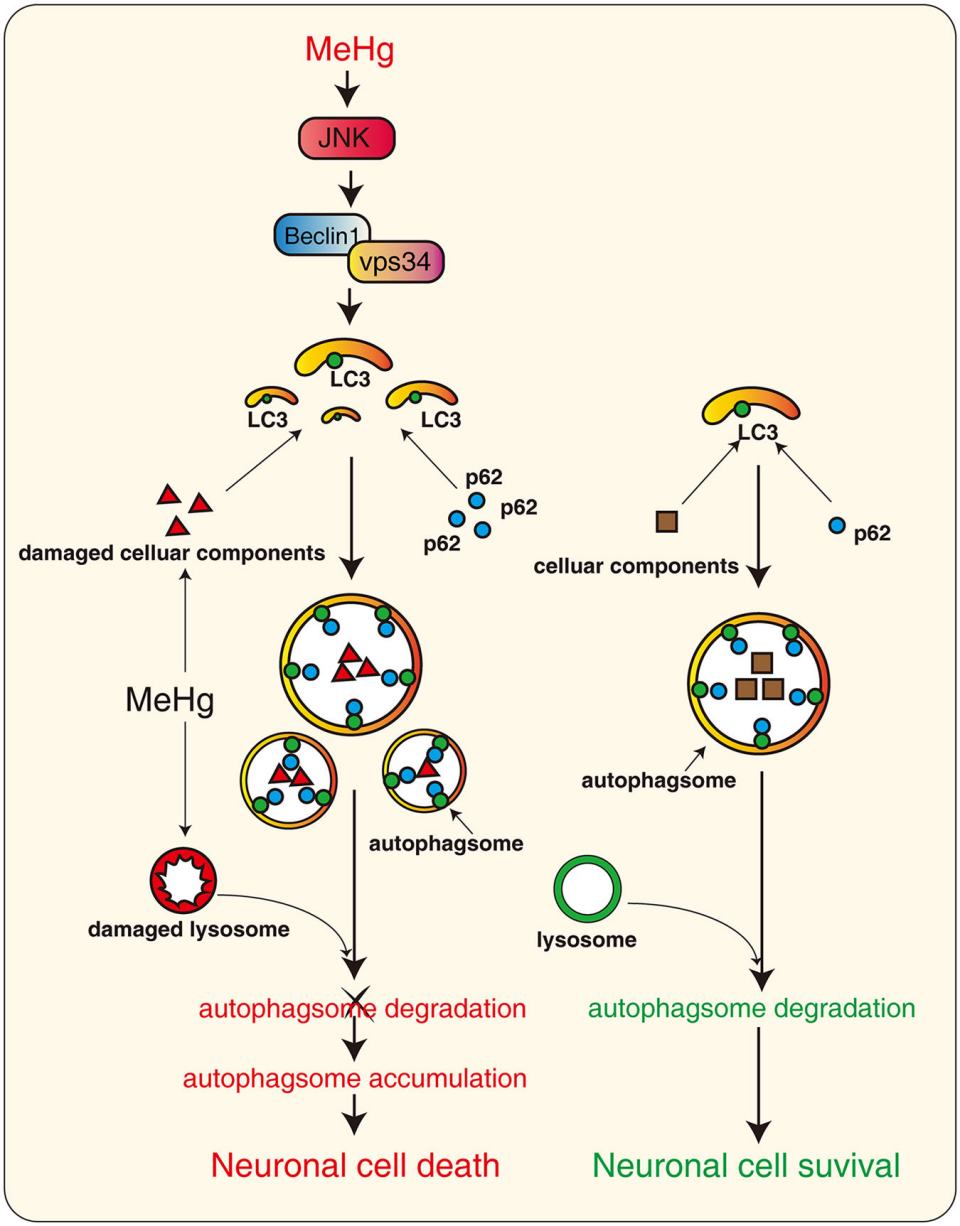
MeHg-activated autophagy induction through the JNK/Vps34 complex pathway elicits autophagosome accumulation and neuronal cell death

In summary, we demonstrated that MeHg-induced autophagy, via JNK/Vps34 complex pathway, promotes autophagosome accumulation and neuronal cells death. These findings would help to understand autophagy in the context of MeHg-induced neurotoxicity (Fig.6), and inhibiting autophagy induction pathway might be a novel therapeutic approach for the treatment of MeHg-induced neurotoxicity.

## Materials and methods

### Cell culture and reagents

Human neuroblastoma cells SH-SY5Y were obtained from the Institute of Biochemistry and Cell Biology (Shanghai, China) and cultured in RPMI 1640 medium supplemented with 10% foetal bovine serum (both Invitrogen, Carlsbad, CA). Primary neuronal cultures were prepared from one-day-old Sprague–Dawley rat. Firstly, cerebral cortex was dissected and chopped, then incubated with 0.125% trypsin/10% EDTA solution at 37 °C for 10 min. The digestion was stopped with FBS. A sterile fire-polished Pasteur pipette was used to triturate the cells gently for about 20 times and then cells were collected through centrifuging at 800 rpm for 5 min. Cells were suspended in DMEM/F12 Medium containing 10%FBS and plated at a density of 2.2 × 10^5^ cells/cm^2^ in plates pre-coated with poli-L-lysine (Sigma–Aldrich, P-1524). DMEM/F12 Medium was changed to Neurobasal Medium (Gibco, A3582901) supplemented with 2% of B27 (Gibco, A3582801), 0.5mM L-glutamine, 100 μg/ml penicillin/streptomycin after 4 h of incubation. The neurobasal medium was refreshed by exchanging half of the volume with fresh media 3 days later. Cells were cultured for 7 days at 37 °C in a humidified 5% CO_2_/95% air atmosphere. MeHg treatment was initiated at 7 days.

MeHg was purchased from Dr.Ehrenstorfer (Germany). Wortmannin, Torin1, SP600125, U0126, SB203580 were purchased from Selleckchem (Houston, TX, USA). LC3 A/B, Beclin1, Vps34, Caspase-9, P-P70s6k Thr389, P-P70s6k Ser371, T-P70s6k, P-4EBP1, T-4EBP1, β-Actin were purchased from Cell Signaling Technologies (Danvers, MA, USA) for western blot. Beclin1, Vps34 were purchased from Bioss (China Beijing) for Immunofluorescence.

### Apoptosis assessment

MeHg-induced apoptosis was measured by flow cytometry using an annexin V-FITC/PI apoptosis detection kit (KeyGEN BioTECH Jiangsu China). Following digestion, the cells were washed twice in ice-cold phosphate-buffered saline (PBS) at a concentration of 5 × 10^5^—1 × 10^6^/ml by centrifugation at 600 × *g* for 5 min and resuspended in 500 µl binding buffer. Then, 5 µl annexin V-FITC and 5 µl propidium iodide (PI) were added and the samples were placed in the dark for 15 min followed by immediate analysis using a FACSCanto II flow cytometer with BD FACSDiva software v6.1.3 (both Becton Dickinson, San Jose, CA). PI as a nuclear and chromosome counterstain that is not permeant to live cells, and annexin V, which binds to the apoptosis marker phosphatidylserine was added to the samples to distinguish necrotic (annexin V−, PI+), late apoptotic events (annexin V+, PI+) from early apoptotic events (annexin V+, PI−). MeHg-induced death of the cerebral cortical neurons was detected using a fluorescent microscope (Nikon ECLIPSE Ti). The cell death rate was calculated as the number of PI+cells/total number of cells.

### Knockdown of Vps34

Four specific siRNAs (small interfering RNAs) against different Vps34 sites were obtained from GenePharma Co. (Shanghai, China) with the following sequences: siRNA-1 sense strand: CACCAAUGAAGCUGAAUAATT, antisense strand: UUAUUCAGCUUCAUUGGUGTT; siRNA-2 sense strand: GGCUGAAACUACCAGUAAATT, antisense strand: UUUACUGGUAGUUUCAGCCTT; siRNA-3 sense strand: CUGGAUAGAUUGACAUUUATT, antisense strand: UAAAUGUCAAUCUAUCCAGTT; siRNA-4 sense strand: GGCAUUGCUUGGAGAUAAUTT, antisense strand: AUUAUCUCCAAGCAAUGCCTT. Scrambled siRNA was used as a negative control (NC) (NC sense strand: UUCUCCGAACGUGUCACGUTT, antisense strand: ACGUGACACGUUCGGAGAATT). The siRNA was introduced into the cells using Lipofectamine RNAiMAX Transfection Reagent (Invitrogen) according to the manufacturer’s instructions.

### Western blot analysis

The proteins were separated by sodium dodecyl sulphate-polyacrylamide gel electrophoresis (SDS PAGE) and transferred onto a PVDF membrane (Millipore Immobilon-FL). The membranes were incubated for 1 h at room temperature in blocking buffer followed by overnight incubation at 4 °C in blocking buffer containing the primary antibody. Then, they were washed three times before incubation with the secondary antibody for 1 h at room temperature. The signal was detected using an Odyssey Infrared Imaging System (LI-COR Biosciences, Lincoln, NE).

### Co-immunoprecipitation (Co-IP)

The cells were cultured in a 100-mm dish. After the designated treatments, they were collected, washed with ice-cold PBS, incubated in lysis buffer for 20 min on ice, and clarified via high-speed (13,000 × *g*) centrifugation at 4 °C for 30 min. The supernatants were incubated overnight at 4 with specific primary antibodies as required followed the addition of 80 µl of Protein G Plus/Protein A Agarose Suspension (Merck Millipore, Darmstadt, Germany) and incubation with gentle rotation at 4 °C for 2 h. The agarose beads were collected and washed five times with lysis buffer and resuspended in 20 ml of 2 × SDS loading buffer. The samples were analysed by western blot.

### Immunofluorescence

For the immunofluorescence studies, 5 × 10^5^ rat cerebral cortical neurons were seeded on a 35-mm confocal plate. After the designated treatments, the cells were washed three times with PBS and fixed in 4% paraformaldehyde for 15 min at room temperature. Then, the cells were passed through frozen methanol for 10 min at −20 °C and blocked in 5% BSA for 30 min. The cells were incubated overnight at 4 °C with the appropriate primary antibody (1:100–1:200) in 5% BSA and with the secondary antibodies (Alexa Fluor^®^ anti-mouse 594 and anti-rabbit 488) (Thermo Fisher) (1:100) in 5% BSA for 60 min at room temperature. An Olympus FluoView™ FV1000 confocal laser scanning microscope with a ×100 objective was used to record the resultant images.

### Adenovirus infection

The cells were infected with the tandem fluorescent-tagged adeno-associated viral vector AAV-mRFP-GFP-LC3 (Hanbio Biotechnology, Shanghai, China) at a multiplicity of infection of 500 and experimentally treated as indicated. This tagged AAV was utilized to observe the intensity of autophagy flux based on the different pH stability of RFP and GFP proteins^26^. The relative fluorescence intensity was detected using an Olympus FluoView™ FV1000 confocal laser scanning microscope with a ×100 objective. GFP, but not mRFP, degrades in an acidic environment. Thus, yellow spots (a mixture of red and green) indicate autophagosomes, whereas red spots indicate autolysosomes (fused phagosome and lysosome). If autophagy is activated and the autophagic flux is normal, the red signal predominates over the yellow signal. If the autophagic flux is impaired, more yellow than red signal will be observed.

### Statistical analysis

Results were expressed as the mean ± standard deviation (S.D.). Student’s *t*-test was used to determine the statistical significance of the difference in values between two groups. One-way ANOVA was used for statistical analysis of the difference in values among multiple groups. *P* < 0.05 was considered significant.

## Supplementary information

Supplemental Figure legends Methods-cell death disease

Supplemental Figure S1

Supplemental Figure S2
