# Supplementary material for: MeHg-induced autophagy via JNK/Vps34 complex pathway promotes autophagosome accumulation and neuronal cell death
Source: Cell Death Dis. 2019 May 21;10(6):399. doi: 10.1038/s41419-019-1632-z (PMC6529499; doi:10.1038/s41419-019-1632-z)
Supplement: Supplementary file 1 — Supplemental Figure legends Methods-cell death disease [file 41419_2019_1632_MOESM1_ESM.docx]

**MeHg-induced autophagy via JNK/Vps34 complex pathway** **promotes autophagosome accumulation and neuronal cell death**

Lin Tianji^1,#^, Ruan Shijuan^1,#^, Huang Dingbang^1^, Meng Xiaojing^1^, Li Wenjun^1^,

Wang bin^1,*^, Zou fei^1,*^

^1^Department of Occupational Heath and Occupational Medicine, School of Public Health, Southern Medical University, Guangzhou, Guangdong 510515, China

# These authors contributed equally to this work.

* Corresponding author. E-mail for Wang bin: [wenwunj@smu.edu.cn](mailto:wenwunj@163.com); E-mail for Zou fei: [zfei@smu.edu.cn](mailto:zfei@smu.edu.cn), Telephone: +86-20-6164-8301, Fax: +86-20-6164-8301.

**Supplemental data**

**Materials and methods**

Cell culture and reagents

Human neuroblastoma cells SH-SY5Y were obtained from the Institute of Biochemistry and Cell Biology (Shanghai, China) and cultured in RPMI 1640 medium supplemented with 10% foetal bovine serum (both Invitrogen, Carlsbad, CA)

MeHg was purchased from Dr.Ehrenstorfer (Germany). U0126, SB203580 was purchased from Selleckchem (Houston, TX, USA). LC3 A/B, Beclin1, Vps34, Caspase-9 , β-Actin was purchased from Cell Signaling Technologies (Danvers, MA, USA) for Western blot. Beclin 1, Vps34 was purchased from Bioss (China Beijing) for Immunofluorescence.

Western blot analysis

The proteins were separated by sodium dodecyl sulphate-polyacrylamide gel electrophoresis (SDS PAGE) and transferred onto a PVDF membrane (Millipore Immobilon-FL). The membranes were incubated for 1 h at room temperature in blocking buffer followed by overnight incubation at 4 °C in blocking buffer containing the primary antibody. Then, they were washed three times before incubation with the secondary antibody for 1 h at room temperature. The signal was detected using an Odyssey Infrared Imaging System (LI-COR Biosciences, Lincoln, NE).

Co-immunoprecipitation (Co-IP)

The cells were cultured in a 100-mm dish. After the designated treatments, they were collected, washed with ice-cold PBS, incubated in lysis buffer for 20 min on ice and clarified via high-speed (13000 g) centrifugation at 4 °C for 30 min. The supernatants were incubated overnight at 4 with speciﬁc primary antibodies as required followed the addition of 80 µl of Protein G Plus/Protein A Agarose Suspension (Merck Millipore, Darmstadt, Germany) and incubation with gentle rotation at 4 °C for 2 h. The agarose beads were collected and washed five times with lysis buffer and resuspended in 20 ml of 2 × SDS loading buffer. The samples were analysed by Western blot.

Apoptosis assessment

MeHg-induced apoptosis was measured by ﬂow cytometry using an annexin V-FITC/PI apoptosis detection kit (KeyGEN BioTECH Jiangsu China). Following digestion, the cells were washed twice in ice-cold phosphate-buffered saline (PBS) at a concentration of 5 × 10^5^ – 1 × 10^6^/ml by centrifugation at 600 g for 5 min and resuspended in 500 µl binding buffer. Then, 5 µl annexin V-FITC and 5 µl propidium iodide (PI) were added and the samples were placed in the dark for 15 min followed by immediate analysis using a FACSCanto II flow cytometer with BD FACSDiva software v6.1.3 (both Becton Dickinson, San Jose, CA).

**Supplemental data**

**Figure legends**

**Fig. S1** MEK inhibitor U0126 and p38 inhibitor SB203580 have no effect on Vps34 complex. **a** SH-SY5Y cells were pretreated with U0126 (10 µM, 1 h), SB203580 (10 µM, 1 h), Then the cells were exposed to 10 µM MeHg for 4 h. The lysates were co-immunoprecipitated using a Beclin 1(Vps34)-specific antibody, and the immunoprecipitants (pull down) were analysed by Western blot for the presence of Vps34 (Beclin1). The whole cell lysates (input) were analysed by Western blot for Vps34 and Beclin 1. **b** SH-SY5Y cells were exposed to 10 µM MeHg for 24 h after preincubation with U0126 (10 µM, 1 h) or SB203580 (10 µM, 1 h). Then, the cells were treated with bafilomycin A1 (baf A1, 400 nM) for 4 h before sample processing. LC3-II was detected by Western blot. All results are representative of three independent experiments.

**Fig. S2** MEK inhibitor U0126 and p38 inhibitor SB203580 have no effect on MeHg-induced neuronal cell death. **a** SH-SY5Y cells were exposed to 10 µM MeHg for 24 h after preincubation with U0126 (10 µM, 1 h) or SB203580 (10 µM, 1 h). Cell apoptosis was detected using an annexin V FITC/PI apoptosis detection kit and flow cytometry analysis. NS＞0.05 versus MeHg group. All results are representative of three independent experiments.
