# Supplementary figures and images for: MeHg-induced autophagy via JNK/Vps34 complex pathway promotes autophagosome accumulation and neuronal cell death
Source: Cell Death Dis. 2019 May 21;10(6):399. doi: 10.1038/s41419-019-1632-z (PMC6529499; doi:10.1038/s41419-019-1632-z)

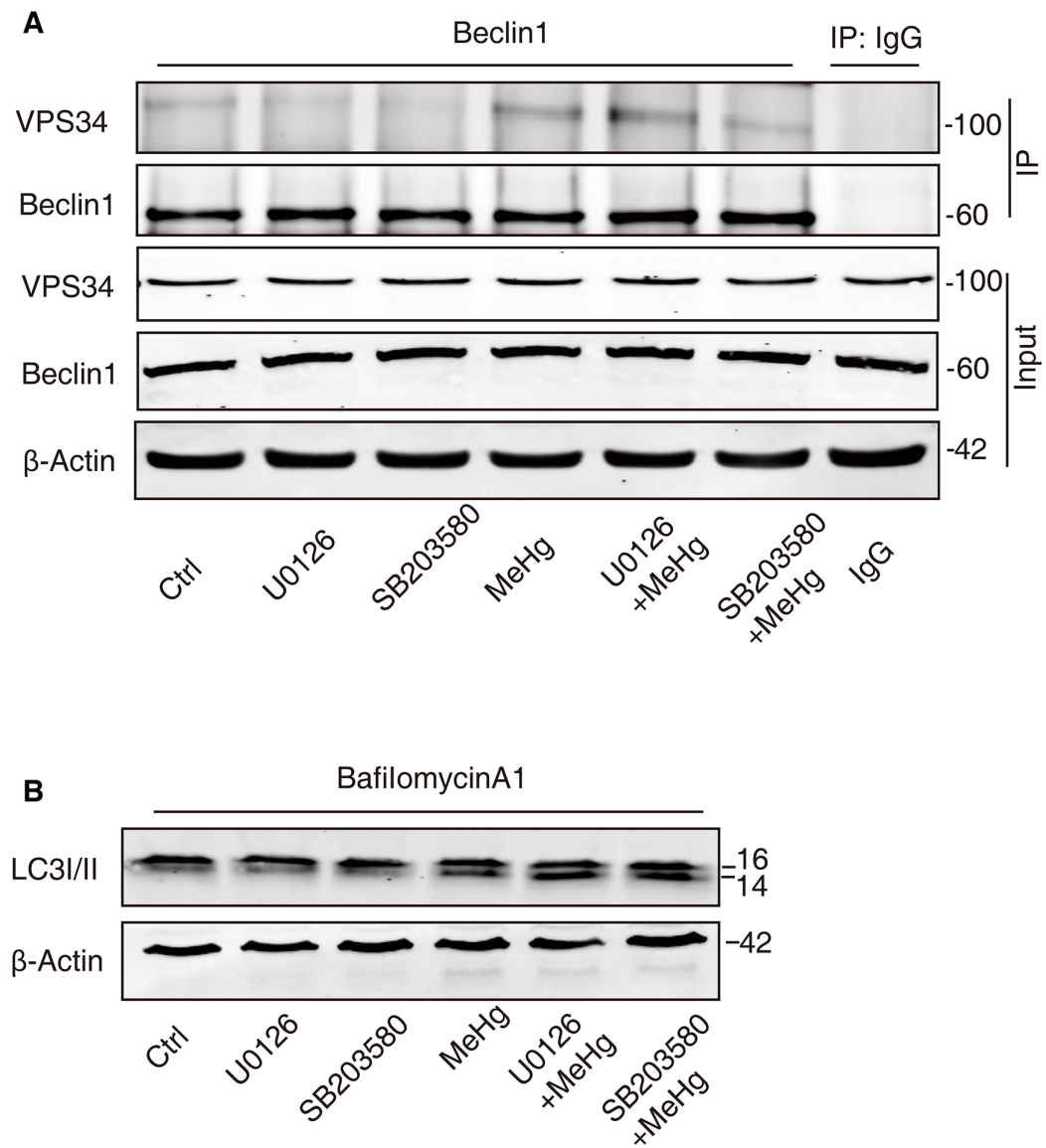

Fig S1

Supplement: Supplementary file 2 — Supplemental Figure S1 [file 41419_2019_1632_MOESM2_ESM.pdf]

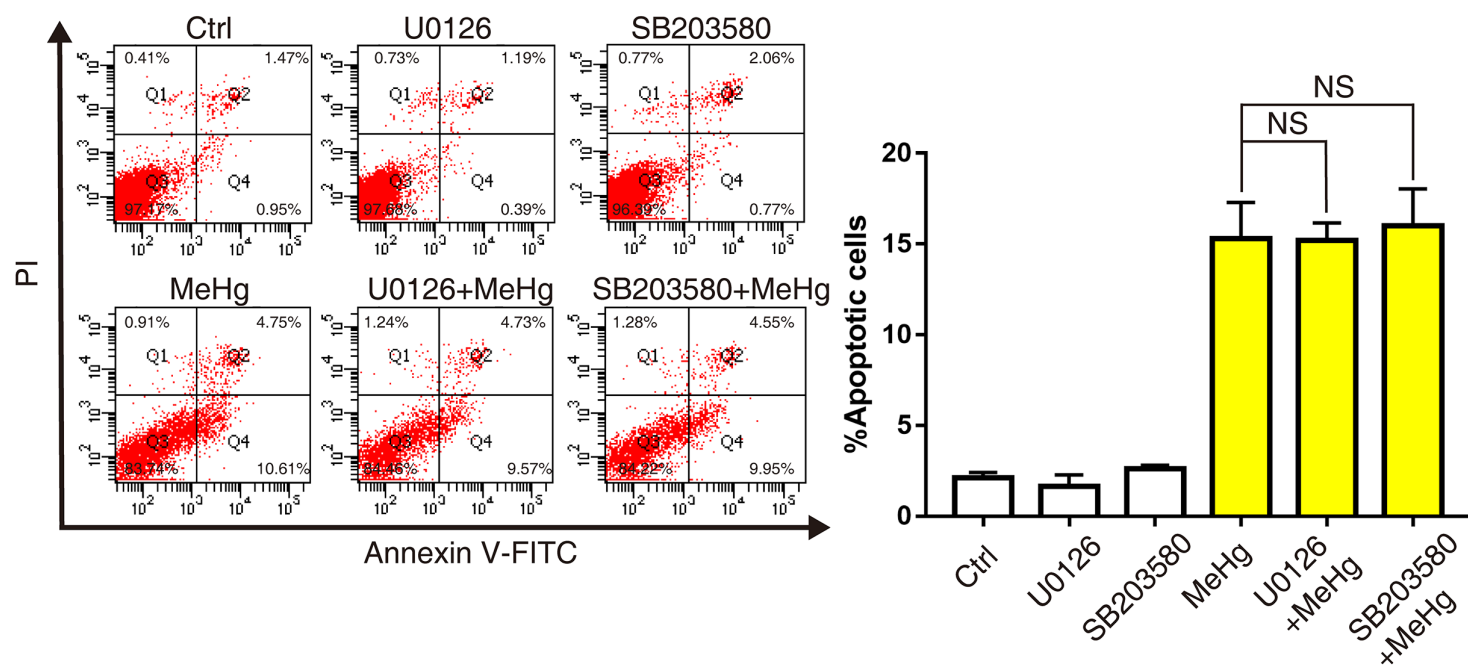

Fig S2

Supplement: Supplementary file 3 — Supplemental Figure S2 [file 41419_2019_1632_MOESM3_ESM.pdf]
